# Prevalence, characteristics, and predictors of healthcare workers with COVID-19 infection in an urban district in Malaysia

**DOI:** 10.11604/pamj.2022.41.243.33300

**Published:** 2022-03-24

**Authors:** Nur Suhada Ramli, Mohd Fadhli Mohd Fauzi, Noor Mohd Amin Moktar, Noriah Hajib, Azmawati Mohammed Nawi

**Affiliations:** 1 Department of Community Health, Faculty of Medicine, Universiti Kebangsaan Malaysia, Jalan Yaakob Latif, Cheras, Kuala Lumpur, 56000, Malaysia,; 2 Pejabat Kesihatan Cheras, Aras 3, Klinik Kesihatan Cheras, Jalan Yaakob Latif, Cheras, Kuala Lumpur, 56000, Malaysia

**Keywords:** Healthcare workers, COVID-19, prevalence, epidemiology, occupational transmission, Malaysia

## Abstract

**Introduction:**

healthcare workers (HCWs) are at high risk of acquiring COVID-19 occupational transmission and subsequently, exposing patients and others. This study aimed to determine the prevalence and examine the characteristics and predictors of HCWs with COVID-19 infection in a Malaysian district.

**Methods:**

this is a cross-sectional study of HCWs working at Cheras District Health Office, with COVID-19 infection from 1^st^ January to 31^st^ October 2021. Data was obtained from the Occupational Safety and Health Unit which included variables of basic sociodemography, type of disease acquisition; healthcare-acquired (HA) or community-acquired (CA), and management outcome. Data was analysed descriptively and cases with type of disease acquisition were compared using logistic regression.

**Results:**

the prevalence of HCWs with COVID-19 was 17.4%. Majority aged 30-39, female gender and Malay ethnicity (51.7%, 60% and 91.7% respectively). Main comorbidities included hypertension (3.3%), diabetes mellitus (3.3%), both hypertension and diabetes mellitus (2.5%) and obesity (4.2%). Smokers, pregnant mothers and non-immunized made up only small proportions (4.2%, 4.2%, and 4% respectively). Paramedics were the most infected proportion (68.4%). About one third of cases managed COVID-19 patients directly (37.5%). Similar proportion had HA infection (29.2%). Smaller proportion (12.8%) needed hospitalization. The early source of infection was HA (January-April). Later, the trend shifted towards CA (May-October). Male gender (OR 3.22, 95% CI = 1.43 - 7.29, p<0.05), smoker (OR 10.84, 95% CI = 1.17 - 100.77, p<0.05), and those who manage COVID-19 cases were more likely to acquire occupational COVID-19 infection (OR 2.28, 95% CI = 1.02 - 5.09, p<0.05).

**Conclusion:**

continuous occupational infectious disease control measures is necessary to reduce the disease burden. Future research on HCWs with COVID-19 infection with larger scale is recommended to determine the final model for predictors of infection.

## Introduction

The coronavirus disease 2019 (COVID-19) pandemic had tremendously affected the world since December 2019. Not only it had impacted the economic and socio-cultural activities, but it also had overwhelmed public health and hospitals capacities in many countries. To a greater extend, health-related effects such as disease infection, mental health burden, and socio-economic effects were among issues faced by the healthcare workers (HCW) while responding to the pandemic healthcare needs [1]. Even more, many of them who have been infected with the severe acute respiratory syndrome coronavirus 2 (SARS-CoV-2), have lost their lives during the pandemic [2]. Healthcare workers are regarded as a high risk group due to their close interactions with infected persons as well as deficiency of personal protective equipment (PPE) during the pandemic time [3-5]. According to the World Health Organization, HCWs is defined as 'all people engaged in actions whose primary intent is to enhance health'. This includes doctors, nurses, midwives, paramedical staff, hospital administrators and support staff and community workers, all of whom now face the occupational risk of becoming infected with COVID-19 [6].

Ensuring the safety and protection of HCWs is a key element in countries´ strategic response to the COVID-19 crisis. Therefore, it is important to understand the epidemiology of COVID-19 infection among HCWs due to its high possible transmission to vulnerable patients, and further depletion of their workforce due to infection can lead to critical shortages, which subsequently cause chaos to healthcare delivery system. Furthermore, for countries to plan for future public health action, it is vital to have proper documentation and analysis of the number of HCWs who have been infected and have died from COVID-19. Nonetheless, it is unclear whether or not data on COVID-19 infection and mortality among HCWs globally are being collected, while those available figures were mostly under-reported [2].

Similarly in Malaysia, although such data are being continuously collected by every Occupational Safety and Health (OSH) Unit in every District Health Office (DHO), its reports remains scarce. Therefore, the aim of this study was to determine the prevalence and examine the characteristics and predictive factors of HCWs with COVID-19 infection in an urban district in Kuala Lumpur. This will provide a snapshot of the situation for HCWs during the current phase of the pandemic, thus enabling focused preventive activities to be made at district levels.

## Methods

This was a cross-sectional analysis of all HCWs working at primary healthcare facilities of Cheras DHO and were tested positive for COVID-19 during the study period.

**Study site:** Cheras is an urban district in Kuala Lumpur, Malaysia. Cheras DHO administrates five main primary healthcare facilities and one public health facility with a total of 690 HCWs employees.

**Study population:** all HCWs with COVID-19 infection from 1^st^ Jan to 31^st^ October 2021, notified to the Communicable Disease Control and Prevention Unit and registered with the OSH Unit of Cheras DHO, were included in this study. These HCWs were tested using the real-time polymerase-chain-reaction (RT-PCR) test if they reported symptoms or were exposed to a confirmed case. A trained OSH doctor investigated, determined the type of acquisition and collected the data required using a standard checklist, following the national guidelines [7]. These data included baseline socio-demographic data, COVID-19 vaccination status, type of disease acquisition and clinical outcomes.

**Operational definition Managing COVID-19 cases directly:** those who manage COVID-19 cases directly would attend to or examine all COVID-19 patients either in the outpatient departments or in the sampling team who were responsible in doing the oropharyngeal or nasal swab test.

**Type of disease acquisition:** Healthcare-acquired (HA) cases refer to either a) HCWs whom source of infection were from patients- considering work or activity during exposure, appropriateness of PPE used during exposure and level of risk of exposure; or b) staff to staff transmission (close contact)- considering activity done during transmission, appropriateness of PPE used during exposure and level of risk of exposure. On the other hand, community-acquired (CA) cases were simply defined as cases whom source of infection were from either their family members, housemates, colleagues, or social interaction.

**Management outcome:** cases who required hospitalization were those with moderate to severe stages of COVID-19 (category 3 or 4 or 5), while cases who received outpatient care were those with mild stage of COVID-19 (category 1 or 2), according to the national guideline [8].

**Sample size calculation:** calculation of sample size for prevalence was done using the Kish formula:


$$n=\frac{Z^{2}P(1-P)}{d^{2}}$$

where n = sample size, Z = Z statistic for a level of confidence, P = expected prevalence or proportion, d = precision. Using prevalence from previous study [9], the sample size needed for this study was 151, for the power of study of 80%. The sample size for predictors were calculated using the PS2 calculator [10] and as follow; male sex (70) [11,12], smoking (290) [13,14], and those managing COVID-19 directly (12) [11,15].

**Data analysis:** data were entered into an Excel spreadsheet and subsequently were transferred into IBM SSPS Statistics version 23 software. Descriptive statistics was used to describe for continuous and categorical data. Univariate analysis was employed to compare HA cases and CA using the Chi-square or Fisher´s exact test, where appropriate. Logistic regression was used to analyze for predictors of HCWs with COVID-19 infection, with binary outcome of type of disease acquisition. A p-value of <0.05 was considered significant. Confidence interval was set at 95%.

**Ethical clearance:** this research was approved by the Medical Research Ethic Committee of Malaysia (NMRR ID-21-02169-2YF).

## Results

During the study period, the prevalence of HCWs infected with COVID-19 at Cheras DHO during the ten-month study period was 17.4% (120 positive cases/690 total HCWs). According to age stratification, majority of infected HCWs were those in the age range of 30-39 (51.7%). This was followed by those in the age group of 20-29 (32.5%), 40-49 (12.5%) and senior age of 50-59 (3.3%). Female gender and Malay ethnicity predominated our study population (60% and 91.7% respectively). Most infected cases did not have comorbidity (81.7%), while among those who had comorbidity, they comprised of hypertension (3.3%), diabetes mellitus (3.3%), both hypertension and diabetes mellitus (2.5%), obesity (4.2%) and others (bronchial asthma and hyperthyroidism) (9.2%). Among all positive cases, only 4.2% were active smokers. From 72 female cases, 4.2% were pregnant. The number of cases who were eligible for COVID-19 vaccination in this study were 99, where the vaccine was only available at Cheras DHO starting from 2^nd^ February 2021 onwards. Out of this, 4% refused for immunization. In our study population, majority were in the group of nurses, medical assistants, attendants, and health inspectors (68.4%), while doctors made up of 18.3% and the remaining were others (drivers, volunteers, lab assistants, and administrative officers) (13.3%). Off all HCWs in this study, about one third (37.5%) dealt and attended to COVID-19 patients directly, and quite a similar portion (29.2%) acquired COVID-19 infection from their healthcare-work place. The majority of cases (87.2%) received outpatient care, while a smaller proportion (12.8%) need to be hospitalized. These socio-demographic characteristics, vaccination status, job and work characteristics, type of disease acquisition and management outcome of HCWs with COVID-19 are shown in Table 1.

**Table 1:** socio-demographic characteristics, vaccination status, job and work characteristics, type of disease acquisition and management outcome of healthcare workers with COVID-19 (N=120)

Variables	Number of HCWs (n)	Percentage (%)
**Age**Age (mean = 33 years)		
20-29 years	39	32.5
30-39 years	62	51.7
40-49 years	15	12.5
50-59 years	4	3.3
**Sex**		
Male	48	40
Female	72	60
**Ethnicity**		
Malay	110	91.7
Non-Malay	10	8.3
**Comorbidity**		
Present	22	18.3
HTN	4	3.3
DM	4	3.3
HTN and DM	3	2.5
Obesity	5	4.2
^a^Others	6	9.2
Absent	98	81.7
**Smoking status**		
Yes	5	4.2
No	115	95.8
^b^ **Being pregnant (N=72)**		
Yes	3	4.2
No	69	95.8
^c^ **Vaccination status (N=99)**		
Yes	95	96
No	4	4
**Job designation**		
Doctor	22	18.3
Nurse/medical assistant/attendant/health inspector	82	68.4
^d^Others	16	13.3
**Managing COVID-19 cases directly**		
Yes	45	37.5
No	75	62.5
**Type of disease acquisition**		
Healthcare-acquired (HA)	35	29.2
Community-acquired (CA5)	85	70.8
^e^ **Management outcome (N=117)**		
Hospitalization	15	12.8
Outpatient care	102	87.2

HCWs = healthcare workers, HTN = hypertension, DM = diabetes mellitus; ^a^Others; bronchial asthma (n=4), hyperthyroidism (n=2); ^b^denominator (N=72) is the number of female cases; ^c^received either first or second dose at time of diagnosis when vaccination programme took place in February 2021; ^d^others; driver (n=5), volunteer (n=5), lab assistant (n=4), administrative officer (n=2); ^e^pregnant cases (n=3) were not included as they must be quarantined at hospital regardless of disease category

**Figure 1:**
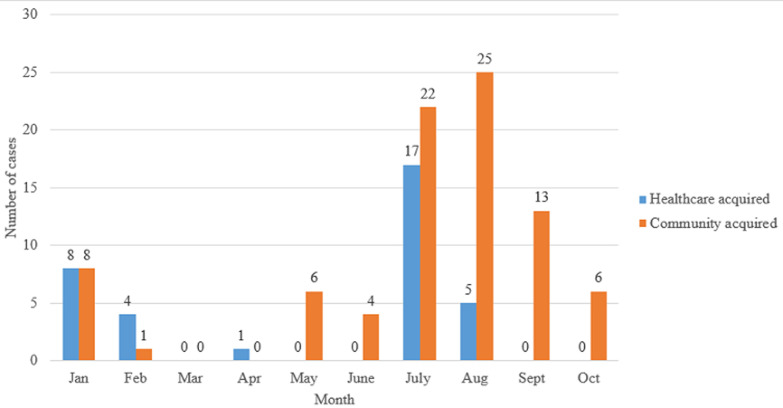
trend of cases according to type of disease acquisition by month

During the initial study period (January to April), the main source of disease acquisition was HA. Later (May to October), the trend shifted towards CA infection. The trend of cases according to type of disease acquisition by month are tabulated in Figure 1. Univariate analysis found there were significant association between some variables studied and type of disease acquisition (Table 2). These included sex (X^2^= 8.26, p <0.05), smoking status (X^2^= 6.53, p <0.05), and managing COVID-19 directly (X^2^= 4.09, p <0.05) (Table 2).

**Table 2:** univariate analysis of the type of disease acquisition and the variables studied

Variable	Type of disease acquisition		Total, n (%)	X^2^	p-value
	HA	CA			
**Age**				4.32	0.229
20-29 years	13	26	32.5		
30-39 years	20	42	51.7		
40-49 years	1	14	12.5		
50-59 years	1	3	3.3		
**Sex**				8.24	*0.004
Male	21	27	40		
Female	14	58	60		
**Ethnicity**				1.94	0.164
Malay	34	76	91.7		
Non-Malay	1	9	8.3		
**Comorbidity**				0.54	0.462
Yes	5	17	18.3		
No	30	68	81.7		
**Smoking status**				6.53	*0.025
Yes	4	1	4.2		
No	31	84	95.8		
**Being pregnant**				0.39	0.535
Yes	1	2	4.2		
No	13	56	95.8		
**Vaccination status**				0.01	0.932
Yes	22	73	96		
No	1	3	4		
**Job designation**			0.242	0.89	
Doctor	6	16	18.3		
Nurse/medical assistant/attendant/health inspector	25	57	68.3		
Others	4	12	13.3		
**Managing COVID-19 cases directly**				4.09	*0.043
Yes	18	27	37.5		
No	17	58	62.5		
**Management outcome**					
Hospitalization	2	13	12.8	2.06	0.151
Outpatient care	32	70	87.2		

HA = healthcare-acquired, CA = community-acquired; X^2^ = Pearson Chi-squared test value; *p<0.05 is significant

Further inferential analysis was able to show for the odds ratio of HCWs with COVID-19 infection according type of disease acquisition. With regard to this matter, the outcome was upon those with HA infection. Similar to findings in Table 2, there were three predictors found this time; being male was three times more likely to get infected with COVID-19 from HA (OR 3.22, 95% CI = 1.43 - 7.29, p<0.05), being smoker was almost eleven times more likely to get infected with COVID-19 from HA (OR 10.84, 95% CI = 1.17 - 100.77, p<0.05), and those who manage COVID-19 cases directly were two times more likely to get infected with COVID-19 from HA (OR 2.28, 95% CI = 1.02 - 5.09, p<0.05) (Table 3). Despite these results, there were no significant findings found in the final model of multiple logistic regression, hence the results are not shown (Table 3).

**Table 3:** logistic regression analysis for predictors of healthcare workers with COVID-19 infection according to type of disease acquisition

Variable	Crude OR	95% CI	X^2^ stat. (df)^f^	p-value^f^
**Age**			5.41 (3)	0.736
20-29 years	1.50	0.14 - 15.88		
30-39 years	1.43	0.14 - 14.61		
40-49 years	0.21	0.01 - 4.48		
50-59 years	1.00			
**Sex**			8.15 (1	*0.005
Male	3.22	1.43 - 7.29		
Female	1.00			
**Ethnicity**			2.33 (1)	0.195
Malay	4.03	0.49 - 33.05		
Non-Malay	1.00			
**Comorbidity**			0.56 (1)	0.464
Yes	0.67	0.23 - 1.97		
No	1.00			
**Smoking status**			5.82 (1)	*0.036
Yes	10.84	1.17 - 100.77		
No	1.00			
**Being pregnant**			0.34 (1)	0.543
Yes	2.15	0.181 - 25.56		
No	1.00			
**Vaccination status**			0.01 (1)	0.932
Yes	1.00			
No	1.11	0.11 - 11.17		
**Job designation**			0.25 (2)	0.886
Doctor	1.13	0.26 - 4.89		
Nurse/medical assistant/attendant/health inspector	1.32	0.39 - 4.48		
Others	1.00			
**Managing COVID-19 cases directly**			4.02 (1)	*0.045
Yes	2.28	1.02 - 5.09		
No	1.00	4		
**Management outcome**			2.35 (1)	0.125
Hospitalization	1.00			
Outpatient care	2.97	0.63 - 13.95		

OR = odds ratio, CI = confidence interval; ^f^likelihood ratio (LR) test

## Discussion

**Prevalence and trend of type of disease acquisition:** in our study, we included a ten-month period prevalence of HCWs infected with COVID-19 to provide an overview of proportion of positive cases in our district healthcare setting. The prevalence of HCWs infected with COVID-19 in our study (17.4%) showed about similar findings from many previous studies done elsewhere (17.1 to 19.4%) [16,17]. Nonetheless, compared with these studies who had all their HCWs underwent the COVID-19 test, only HCWs with symptoms or who became close contacts underwent for COVID-19 RT-PCR test in our study. In some other countries, fewer percentage were also reported (5.6 to 10.6%) [18-20].

At the beginning of the study period (Figure 1), there were more cases whose disease acquisition was HA. During that time, although there was adequate supply of PPEs, there were also evident of high prevalence of burnout among HCWs in Malaysia [21-23]. This psychological distress may further deteriorates the health condition of the already exhausted HCWs, hence exposing them to higher risk for COVID-19 infection. Later in the timeline, the trend of disease acquisition shifted towards CA, and was more prominent in May to October, and the trend was consistent with the soaring number of positive cases nationwide that reached peak in the month of August during the fourth-wave of COVID-19 pandemic that hit Malaysia [24]. In general, our finding showed there were more HCWs who acquired COVID-19 infection from the community as compared to their healthcare work place. This was also consistent with results from other studies done elsewhere [18,25].

**Characteristics of HCWs with COVID-19 infection:** the mean age of infected HCWs in this study was 33 years and this was almost consistent in other studies [20,26]. This owes to the fact that most HCWs who were stationed at frontline healthcare facilities were in this young age category. Our result showed there were more female HCWs who were infected with COVID-19 (60%) as compared to male. Similar findings were also reported by other studies [12,20,26,27]. The high proportion of Malay ethnicity infected (91.7%) was relatively proportionate with their numbers in the entire Cheras DHO organization. Majority (81.7%) of the infected HCWs had no chronic diseases, possibly due to the high proportion of young age of our study population. Among those with comorbid (18.3%), common ones included obesity, diabetes mellitus, and hypertension, where these three conditions are known to be risk factors for COVID-19 infection [28-30]. Very small proportion were smokers and pregnant (both were 4.2%). These two conditions are also established risk factors for COVID-19 infection [31,32], thus extra caution must be taken by them.

Although the COVID-19 vaccination may protect an individual from getting infected, the risk of getting infected still exists. Among HCWs who have completed their vaccine, the occurrence of the SARS-CoV-2 infection was found to be related with the neutralizing antibody titers during incubation period [33]. While most infections appeared asymptomatic to mild, persistent symptoms did occur [33]. Therefore, those who did not had COVID-19 vaccination in our study population (4%) were given health education regarding the importance of getting the jab. In most settings, paramedics are the ones who spend most of their time treating and handling patients, hence putting themselves at the highest risk of getting any disease infection. This explains the occurrence of their highest percentage (82%) as shown in this study, where this was also agreed by other studies [9,27].

The low hospitalization rate (12.8%) in our study population was about the same as previous studies done in other countries [18,26], since most had only mild to moderate infection that did not require admission. Higher proportion of infected HCWs in our study population were posted in the low-risk zones (62.5%) and did not manage COVID-19 cases directly. Interestingly, our result contrasted from results of other studies [20,34] where higher percentage were seen among those working in the-high risk zones. According to a study, such a scenario occurred mainly when COVID-19 was not suspected in patients and no adequate PPE was worn (Nienhaus and Hod 2020).

**Predictors of HCWs with COVID-19 infection from occupational transmission:** three predictors for HCWs who acquired infection from their healthcare workplace were found in this study. Our study found that male HCWs were more likely to get HA infection as compared to female HCWs. According to a study of gender difference during the current COVID-19 pandemic, male HCWs paid less attention and presented with lower anxiety, lower self-evaluation with hand hygiene, wearing gloves and surgical mask [35]. While in general population, they were three times more likely to be admitted into the intensive treatment unit and have higher mortality compared to females [36]. Secondly, our study showed that HCWs who smoke carried higher odds to get HA work-related infection. As SARS-CoV-2 transmits through salivary droplets, tobacco smokers are also at higher risk of COVID-19 infection due to poor lung function, cross-infection and susceptible hygiene habits [37]. In addition, patients who were previously smoked were more likely to develop more severe symptoms of COVID-19 disease than non-smokers, while data on COVID-19 prevalence among smokers and non-smokers is thus far, still inconclusive [38]. Finally, HCWs who manage COVID-19 cases directly are known to have higher risk to get occupational transmission infection, as shown in our study. On average, as high as 80% of inhalation exposure happens when HCWs are stationed close to patients [39]. Relatively, air droplets and inhalation depends upon the SARS-CoV-2 emission in respirable particles through infected persons´ exhaled breath, and viral inhalation are most likely to occur when their emission exceeds five gene copies per minute [39]. Thus being close and managing COVID-19 cases directly put HCWs at higher odds for HA.

**Strength and limitation:** to our knowledge, this was the first study done on HCWs with COVID-19 infection in an urban DHO in a developing Asian country. Therefore, the findings provide an overview of the impact of current COVID-19 pandemic towards our HCWs in primary settings. Despite the findings, our data was lacking from its calculated sample size, as we encountered difficulties to include previous data due to poor documentation, hence hindering significant results from the final multiple logistic regression model. Since study setting was done at primary healthcare settings, generalization of results cannot be made to other level of healthcare settings.

## Conclusion

The strengthening of infective control measures to prevent occupational transmission of COVID-19 is necessary to reduce the disease burden. The documentation of infectious disease occurrence is important towards public health preparedness for disease prevention. Examples of such activities include robust surveillance, periodic auditing of all HCWs and relevant systems, and continuous health education. All HCWs should always be vigilant and take extra caution in current pandemic era, by abiding to all precaution measures. Also, future research on HCWs with COVID-19 infection with larger scale is recommended to determine the final model for predictors of infection according to type of disease acquisition.

### What is known about this topic

- Until today, there were limited data on COVID-19 infection and death among HCWs globally.

### What this study adds

- To our knowledge, this was the first study done on epidemiology of HCWs with COVID-19 infection in an urban district in a developing Asian country;
- The findings provide an overview of the impact of current COVID-19 pandemic towards our HCWs in primary settings.
